# What determines the interfacial configuration of Nb/Al_2_O_3_ and Nb/MgO interface

**DOI:** 10.1038/srep33931

**Published:** 2016-10-04

**Authors:** J. L. Du, Y. Fang, E. G. Fu, X. Ding, K. Y. Yu, Y. G. Wang, Y. Q. Wang, J. K. Baldwin, P. P. Wang, Q. Bai

**Affiliations:** 1 State Key Laboratory of Nuclear Physics and Technology, School of Physics, Peking University, Beijing 100871, P. R. China; 2 State Key Laboratory for Mechanical Behavior of Materials, Xi’an Jiaotong University, Xi’an, 710049, P. R. China; 3 Department of Materials Science and Engineering, China University of Petroleum, Beijing 102249, P. R. China; 4 Experimental Physical Sciences Directorate, Los Alamos National Laboratory, Los Alamos, NM 87544, USA

## Abstract

Nb films are deposited on single crystal Al_2_O_3_ (11
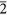
0) and MgO(111) substrates by e-beam evaporation technique. Structure of Nb films and orientation relationships (ORs) of Nb/Al_2_O_3_ and Nb/MgO interface are studied and compared by the combination of experiments and simulations. The experiments show that the Nb films obtain strong (110) texture, and the Nb film on Al_2_O_3_(11
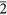
0) substrate shows a higher crystalline quality than that on MgO(111) substrate. First principle calculations show that both the lattice mismatch and the strength of interface bonding play major roles in determining the crystalline perfection of Nb films and ORs between Nb films and single crystal ceramic substrates. The fundamental mechanisms for forming the interfacial configuration in terms of the lattice mismatch and the strength of interface bonding are discussed.

Heterophase interfaces play an important role in determining the physical properties of materials from multiple aspects^1^,^2^,^3^,^4^,^5^,^6^,^7^,^8^,^9^. For example, it is well-known that electrical resistivity can be tuned by interfaces which usually act as obstacles to charge carriers^10^. More over, it has been suggested that the density and atomic structure of dissimilar interfaces underpin the mechanical and physical properties of the materials by exerting influence on nucleation and slip of dislocations, the kinetics of charge carriers and so on^11^,^12^,^13^,^14^,^15^,^16^. Extensive studies on metallic multilayers have demonstrated that the strength of multilayers increases with decreasing monolayer thicknesses while the total thicknesses of the films remain constant^17^,^18^.

Metal/oxide interfaces are of immense interest as their atomic structures are different from metal/metal counterparts. With the presence of an oxide constituent, the bonding of interface is changed from the delocalized metallic bonding to ionic or covalent bonding^19^,^20^. Experiments indicate that the special ORs with low-indexed planes between metal and oxide crystal exist in metal/oxide interface^21^. It has been suggested that the crystallographic ORs between the metal film and oxide substrate, especially the bonding between the first layer metal atoms and oxide substrate layer at the interface, determine the properties of metal films^22^,^23^,^24^,^25^. Experiments show that the ORs of interfaces are largely determined by lattice mismatch^20^,^26^, e.g. Nb^27^,^28^, Fe^22^,^29^, V^30^,^31^, Ta^32^, and Cr^20^ on MgO(100) substrates. However, it is also known that a particular metal/oxide interface with similar lattice mismatch has two or more ORs, in which the lattice mismatch alone is insufficient to explain the formation of the ORs. Previous calculations show that the ORs between metal film and oxide substrate are dominated by the of interface bonding^25^,^27^,^33^. So it is not clear about the roles that the lattice mismatch and the strength of interface bonding play in determining the interfacial configuration of metal/ceramic interface.

Featured by the high immiscibility and excellent thermal expansion compatibility, high purity single-crystal Al_2_O_3_ and MgO are widely used as substrates for Nb thin films^34^. Most of the studies of Nb/Al_2_O_3_ interface are focused on the Nb(111)/Al_2_O_3_(0001) interfaces and these interfaces are found to be semicoherent, showing coherent regions separated by misfit dislocations^34^,^35^. As for Al_2_O_3_(11
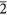
0) substrate, Nb film exhibits Nb(110) texture^36^. Also, our previous study showed that the Nb/MgO system exhibits a preferential orientation of Nb(110) plane in parallel to the (111) surface of the MgO (111) substrate^27^. Thus, Nb films deposited on both substrates show the preferred orientation of Nb (110).

In this article, the Nb films were epitaxially grown on Al_2_O_3_(11
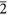
0) and MgO(111) substrates, respectively, and the fundamental mechanisms for the formation of interfacial configuration were systematically investigated by experiments and simulations. Comparison of the two systems, in terms of crystalline perfection of Nb film and interface orientation, indicates that the lattice mismatch and the strength of interface bonding are dominating factors for the formation of Nb/oxide ORs.

## Results

**Figure 1:**
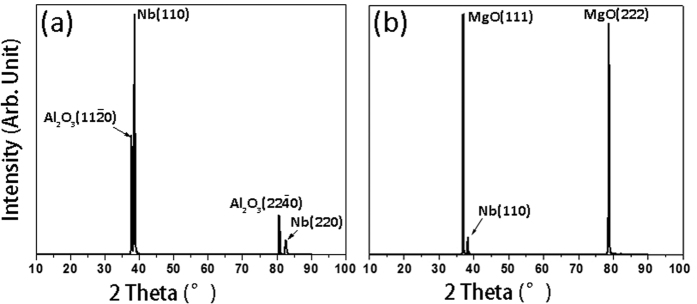
XRD results of (**a**) Nb film on Al_2_O_3_ (11
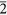
0) substrate and (**b**) Nb film on MgO(111) substrate showing strong Nb (110) texture in both cases.

Nb is a body centered cubic (BCC) crystal with the lattice parameter of 0.3307 nm. Al_2_O_3_ is a hexagonal close packed (HCP) crystal with the lattice parameters of a = 0.4759 nm and c = 1.2991 nm, and MgO has a face centered cubic (FCC) structure with the lattice parameter of a = 0.4217 nm. Figure 1a shows XRD θ–2θ diffraction pattern of the Nb film on Al_2_O_3_ (11
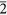
0) substrate. The specimen shows a strong diffraction peak of Nb(110) together with a peak of Al_2_O_3_(11
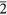
0), and a weak diffraction peak of Nb(220) together with a peak of Al_2_O_3_(22
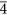
0). No other diffraction peaks are present, indicating that the Nb film is strongly textured wherein the Nb(110) plane is parallel to the Al_2_O_3_(11
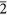
0) plane. Figure 1b shows the XRD θ–2θ diffraction pattern of Nb film on MgO(111) substrate. Despite the deposition substrate difference, both Nb film on Al_2_O_3_(11
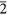
0) and Nb film on MgO (111) exhibit the strong texture of Nb (110) as evidenced by XRD results.

The interplanar spacing *d* of Nb (110) can be obtained from the Bragg’s law:


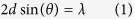


where λ = 1.5406 Å in this experiment. The peak position of Nb(110) in experimental plots is calibrated by diffraction peak of Al_2_O_3_(11
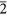
0) and diffraction peak of MgO(111), respectively. If the sample presents uniform strain *ε*′ at the interface, the modified interplanar spacing of Nb(110) will produce a peak shift to a new position. The uniform strain *ε*′ is defined as:


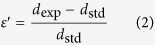


where *d*_exp_ and *d*_std_ are the experimental and standard interplanar spacing of Nb(110). The out-of-plane interface uniform strains of Nb/Al_2_O_3_ system and Nb/MgO system are −0.005 and 0.004, respectively. The positive and negative signs represent the expansion and contraction of lattice along the out-of plane direction. The full width at half maximum (FWHM) values of the Nb(110) diffraction peak are 0.226° and 0.556° for Nb/Al_2_O_3_ and Nb/MgO system respectively. Since the peak broadening typically arises from the non-uniform strain, it indicates that the Nb film grown on MgO(111) substrate possesses a larger non-uniform strain than that grown on Al_2_O_3_(11
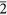
0) substrate.

**Figure 2:**
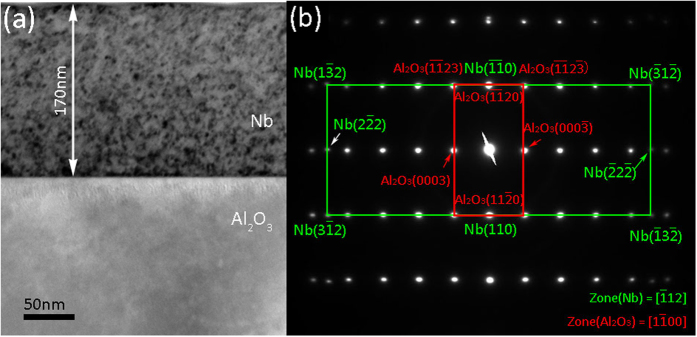
(**a**) Bright-field TEM micrograph of Nb(110)/Al_2_O_3_(11
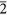
0). (**b**) Corresponding selected area diffraction (SAD) pattern shows the film exhibits single-crystal like structure and well-defined orientation relationships of interface.

Figure 2a is the bright field TEM micrograph of Nb film on Al_2_O_3_(11
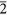
0) substrate in the zone axis of Al_2_O_3_[1
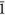
00]. The Nb (110)/Al_2_O_3_ (11
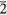
0) interface is sharp and flat. Figure 2b shows selected area diffraction (SAD) pattern of the interface, which reveals that the ORs between Nb film and Al_2_O_3_ substrate are Nb(110)/Al_2_O_3_(11
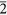
0), Nb[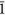
12]//Al_2_O_3_[1
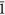
00] and Nb[1
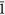
1]//Al_2_O_3_[0001].

Based on the ORs above, the lattice mismatch along these two directions can be calculated by:


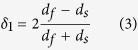


where *d*_f_ and *d*_s_ are the plane spacing of the bulk Nb lattice and the substrate respectively^37^. In this paper, the lattice mismatch which is observed along the Al_2_O_3_[1
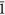
00] and Nb[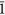
12] is calculated as *δ*_1_ = 12.5%. Another lattice mismatch is observed along the crystal orientation Al_2_O_3_[0001] and Nb[1
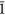
1], which is perpendicular to the crystal orientation of Al_2_O_3_[1
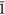
00]. The lattice mismatch is calculated to be *δ*_2_ = 1.8%.

**Figure 3:**
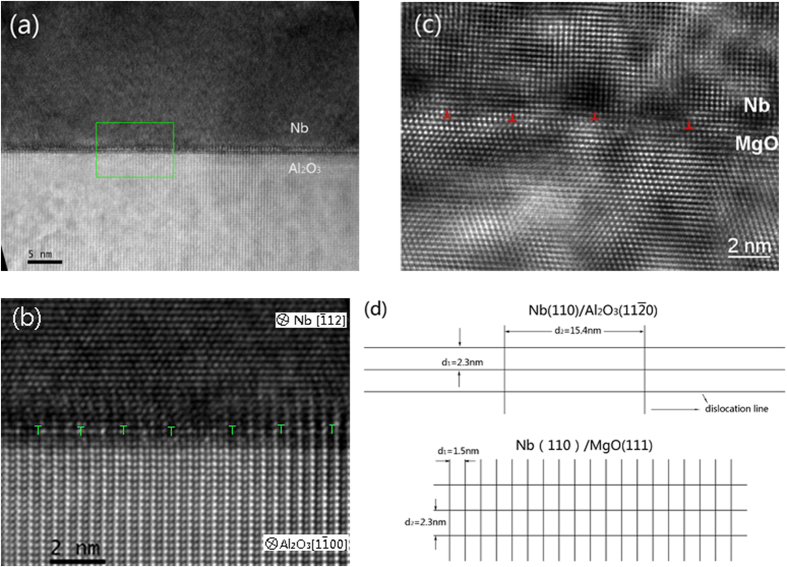
(**a**) HRTEM micrograph of Nb(110)/Al_2_O_3_(11
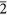
0) interface observed along Al_2_O_3_[1
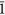
00] zone axis. (**b**) Enlarged image of the region labeled by the box in (**a**). (**c**) HRTEM micrograph of Nb(110)/MgO(111) observed along MgO[1
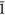
0] zone axis. (**d**) Schematics of dislocation lines’ networks of Nb(110)/Al_2_O_3_(11
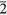
0) interface (up panel) and Nb(110)/MgO(111) interface (bottom panel).

High resolution TEM (HRTEM) image of the Nb/Al_2_O_3_ interface is shown in Fig. 3a,b. An array of misfit dislocations is observed, which are labeled by the “T” sign in Fig. 3b. The average spacing of misfit dislocations was measured to be 1.9 nm. Also, the average interface dislocation spacing can be given by^36^,^37^,^38^:


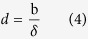


where b is the Burgers vector’s absolute value of the misfit dislocation parallel to the interface and *δ* is the lattice mismatch. By using eq. (4), the average interface dislocation spacing is calculated as *d*_1_ = b/*δ*_1_ = 2.3 nm, where the Burgers vector in Nb(110)/Al_2_O_3_(11
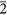
0) system parallel to the interface is **b** = 1/2Nb[1
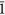
1]. The measured average spacing between dislocations is 1.9 nm, similar to the calculated value of 2.3 nm. It shows that the lattice mismatch *δ*_1_ calculated by eq. (3) is close to the real interface. The spacing between dislocations, which is observed along the crystal orientation Al_2_O_3_[0001] and Nb[1
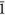
1] is *d*_2_ = b′/*δ*_2_ = 15.4 nm, where the Burgers vector parallel to the interface and perpendicular to the Nb[1
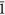
1] is **b**′ = 1/3Nb[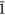
12]^34^.

Figure 3c shows the HRTEM micrograph of Nb/MgO interface observed along the MgO[1
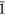
0]. The ORs were found as Nb(110)//MgO(111), Nb[001]//MgO[1
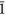
0] and Nb[1
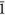
0]//MgO[11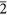], thus the lattice mismatch along two in-plane directions can be calculated as *δ*_1_ = 10.1%, which is observed from the MgO[11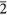] direction, and another lattice mismatch which is observed from the MgO[1
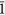
0] direction is *δ*_2_ = 10.2%. By using eq. (4), the corresponding dislocation spacing between misfit dislocations is *d*_1_ = 1.5 nm and *d*_2_ = 2.3 nm.

Based on the above calculations, the planar dislocation lines’ networks of Nb(110)/Al_2_O_3_(11
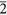
0) and Nb(110)/MgO(111) are drawn in Fig. 3d. The difference in the calculated dislocation spacing along the interface implies the difference of the dislocation density along the interface. The dislocation density of Nb(110)/Al_2_O_3_(11
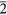
0) interface is lower than that of Nb(110)/MgO(111) interface. As a result, the Nb(110)/Al_2_O_3_(11
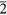
0) interface has less distortion and thus it is more stable than Nb(110)/MgO(111) interface.

In short, the ORs of Nb/Al_2_O_3_(11
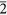
0) and Nb/MgO(111) interfaces are observed by TEM. The lattice mismatches along two perpendicular in-plane directions for Nb/Al_2_O_3_ interface are 12.5% and 1.8%, while for Nb/MgO interface are 10.1% and 10.2%. HRTEM analysis shows that the measured lattice mismatch is consistent with the calculated value.

Above study shows that both Nb film on Al_2_O_3_(11
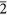
0) and Nb film MgO (111) exhibit the strong texture of Nb (110) plane, and it is essential to investigate the films crystalline perfection which can be characterized by channeling RBS and XRD rocking curve.

**Figure 4:**
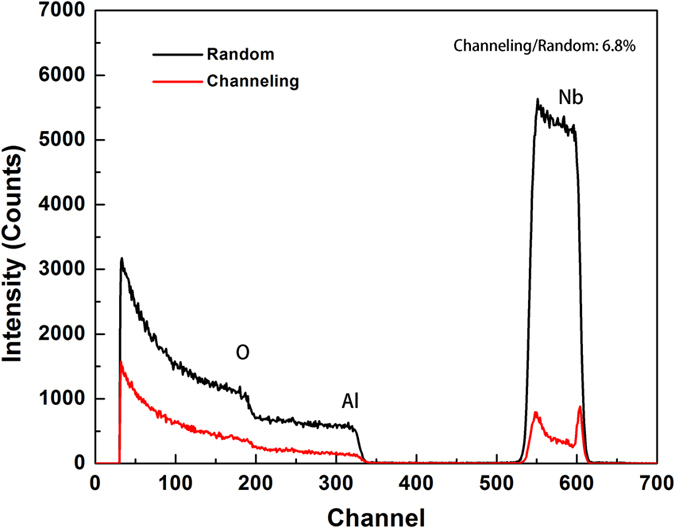
**2.0 MeV** ^**4**^**He**^**+**^ **RBS/Channeling spectra of Nb(110) film on Al**_**2**_**O**_**3**_**(11**
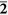
**0) substrate.** The thickness of Nb film is about 170 nm. The aligned spectrum was taken along the [110] axis of the Nb film.

Figure 4 illustrates 2 MeV ^4^He^+^ RBS spectrums from Nb(110) film on Al_2_O_3_ (11
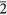
0) substrate taken under random and [110] aligned condition. The yield ratio of the aligned spectrum to random spectrum was used to characterize the crystalline perfection along the out-of-plane direction. The peaks of random spectrum line (black line) and aligned spectrum line (red line) correspond to Nb, Al and O atoms with the decrease of the channel, respectively. The aligned spectrum has two shoulders on the Nb peak. The peak at the higher channel side is the surface peak, mainly from the direct collisions of He ions with the surface Nb atoms in the film. The peak at the lower channel side reflects the imperfection of crystal lattice of Nb film near the Nb(110)/Al_2_O_3_(11
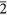
0) interface^39^,^40^,^41^. The yield ratio of the Nb[110] aligned spectrum to random spectrum from Nb at the position just below the surface peak is about 6.8%, demonstrating that the Nb film deposited on Al_2_O_3_ substrate has a well aligned crystalline orientation. The same experiments of Nb(110)/MgO(111) interface was done, however, it was too difficult to observe the channeling effect, indicating that the Nb(110) film on MgO(111) substrate has highly mis-oriented crystallites. These results indicate that the crystalline quality of Nb film deposited on MgO(111) substrate is worse than that deposited on Al_2_O_3_(11
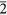
0).

**Figure 5:**
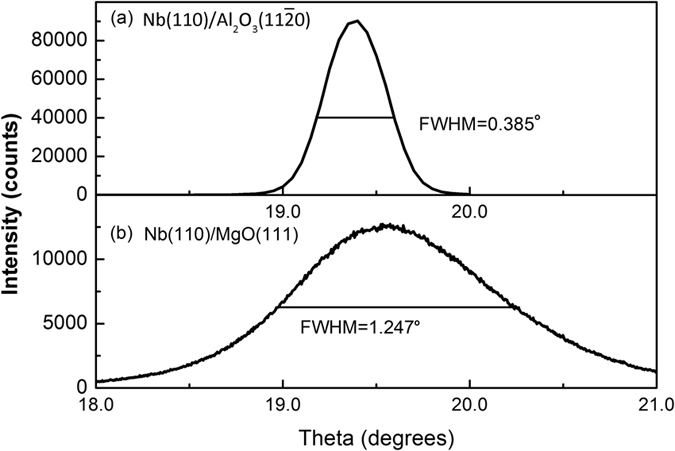
The rocking curves of Nb films on (**a**) Al_2_O_3_(11
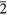
0) substrate and (**b**) MgO(111) substrate characterizing the crystalline quality of Nb films.

High resolution X-ray diffraction is used to investigate the crystalline quality of films and evaluate the dislocation density of interfaces^42^,^43^,^44^,^45^,^46^. The measured full width at half maximum (FWHM) from the XRD rocking curves is obtained by rotating the sample through the Bragg angle after the angle (θ) and the detector position (2θ) are fixed at the Bragg angle of the corresponding reflection. The FWHM is used to determine the mean spread (range) in orientation of the different out-of-plane crystalline domains of a perfect crystal with mis-orientation. Defects like mosaicity, misfit dislocations and mis-orientation will create disruptions in the perfect parallelism of atomic planes and thus broaden the width of rocking curve. Figure 5 illustrates the rocking curves of Nb thin films on Al_2_O_3_(11
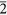
0) substrate (Fig. 5a) and MgO(111) substrate (Fig. 5b). The peaks of both Fig. 5a,b are reflected from Nb(110) lattice planes. The FWHM of the rocking curve is a way to quantify how well the crystallites in the film are ordered along the growth direction, where in general smaller FWHM values indicate better ordering along the growth direction^47^. Measured FWHM for Nb film deposited on Al_2_O_3_(11
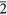
0) plane is 0.385°, while the value for Nb film deposited on MgO(111) is 1.247°. Thus Fig. 5 indicates that the atomic alignment along Nb(110) direction in the Nb(110)/MgO(111) film is significantly worse than that in the Nb(110)/Al_2_O_3_ (11
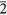
0) film, consistent with the conclusion from the RBS/Channeling measurements.

## Discussion

### Comparison of the degree of crystalline perfection in terms of defect density

XRD and TEM studies show that the Nb films with preferred orientation of plane (110) were grown on Al_2_O_3_(11
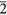
0) and MgO(111)substrates. The comparison of XRD rocking curves and RBS/Channeling measurements of Nb(110)/Al_2_O_3_(11
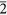
0) and Nb (110)/MgO(111) shows that Nb(110) film grown on Al_2_O_3_ (11
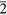
0) substrate has higher crystalline quality. Film with higher crystalline quality usually has lower defects, and often dislocations are thought to be the main defects in the epitaxial single crystal thin film. Here the dislocation density of Nb films will be estimated by the FWHM values of rocking curves through the Hirsch model using equation^45^,^48^:


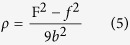


where F is the width of the rocking curve (FWHM) in radians, *f* is the FWHM in radians of monochromator, and *b* is the magnitude of the Burgers vector in cm. In present experiment, *f* is small compared to the measured rocking curve width of our samples and can be negligible. The Burgers vector of Nb thin film is **b** = 1/2Nb[1
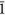
1] in this paper and thus the dislocation density of Nb film grown on Al_2_O_3_(11
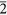
0) substrate is 
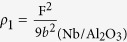

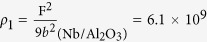
/cm^2^ and the dislocation density of Nb film grown on MgO(111) substrate is 
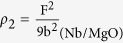

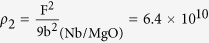
/cm^2^. As expected, the Nb/MgO film contains much greater dislocation density than the Nb/Al_2_O_3_ film.

To further understand the difference of crystalline quality, the lattice mismatch of these two interfaces is investigated. It is well known that interface with larger lattice mismatch usually leads to smaller misfit dislocation spacing. The interface misfit dislocation intersections (MDIs) in interface are usually used to characterize the interface dislocation lines’ network^49^,^50^,^51^,^52^ and the areal density of MDIs is the indication of the misfit dislocation density. From the dislocation spacing of the two perpendicular directions which are parallel to interface, the areal density of MDIs can be defined as:


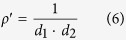


where 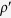 is the dislocation density of misfit dislocation, *d*_1_ and *d*_2_ are the dislocation spacings which are perpendicular with each other and can be obtained from Eq. (4). The areal density of MDIs of Nb(110)/Al_2_O_3_(11
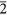
0) interface is 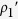 = 2.8 × 10^12^/cm^2^, while the areal density of MDIs of Nb(110)/MgO(111) interface is 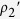 = 2.9 × 10^13^/cm^2^. This result shows that the MDIs density in Nb/Al_2_O_3_ system is much smaller than that in Nb/MgO system, thus the misfit dislocation density of Nb/Al_2_O_3_ interface is lower than that of Nb/MgO interface.

Commonly, the occurrence of misfit dislocations in epitaxial films, which further lowers the crystalline quality of single crystal films, is contributed to the decrease of film’s strain energy before and after the formation of misfit dislocation. Strain energy change in thin film with the thickness (*h*) can be written as 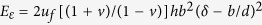, where *u*_*f*_ and *v* are the shear modulus and poisson’s ratio of film. Clearly, only the term (*δ* − *b*/*d*)^2^ is related to the strain energy difference between Nb film grown on MgO and on Al_2_O_3_ substrate. The Nb film grown on MgO substrate has a larger lattice mismatch (*δ*) and a larger dislocation spacing (*d*), thus has a larger decrease of strain energy than that grown on Al_2_O_3_ substrate, therefore, leading to more dislocations in Nb film in Nb/MgO. As a whole, the Nb/MgO interface has larger lattice mismatch and thus leads to higher density misfit dislocation in the interface. Subsequently, the stress fields of misfit dislocations promote the occurrence of defects during the following film growth. As a result, Nb/MgO has low crystalline quality of Nb film.

### Orientation relationships of Nb/Al_2_O_3_ and Nb/MgO interface

In order to understand the fundamental mechanisms that why these interfacial configurations behave as they are, the first principle calculation through the coherent models was used to understand their orientation relationships. The building process of the interface structure models is shown in Support Information.

Work of separation (*W*_*Sep*_), defined as the reversible energy needed to separate the interface into two free surfaces, is a fundamental quantity to characterize the strength of the metal/oxide interface, thus it was calculated for the six models. *W*_*sep*_ is calculated as^25^:


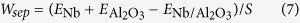


where *E*_Nb_, 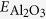 is the energy of single Nb slab and single Al_2_O_3_ slab, respectively, 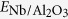 is the total energy of the whole coherent structure after energy minimization, and *S* is the interface area. Two preset cutoff values of lattice strain (*ε*) and interface mismatch (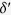) are used to reduce the number of interfacial configuration models. The lattice strain is the strain of Nb along a given direction, e.g., *ε*_*l*_ = (*a*′ − *a*)/*a*, where *a*′ and *a* are the lattice constants after and before deformation along *l* direction. The interface mismatch (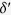) refers to the strain required to make two unit cells form coherent structure, and is defined as 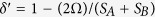, where Ω represents the overlap area of unit cell with the match cell area of *S*_*A*_ in the film and the match area of *S*_*B*_ in the substrate. It should be noted that the 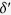 is commonly used to describe two plane mismatch of heterointerface, different from the lattice mismatch *δ* in an axial direction. Study shows that there are six potential models of the Nb/Al_2_O_3_(11
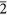
0) system and these models are shown in Figure S1b. The calculation results for the six potential models are shown in Table 1.

In addition, we also calculated the atomic Mulliken population to check the information about electron transfers along the interface. For the six Nb/Al_2_O_3_(11
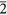
0) coherent structures, we focused on the difference of Mulliken population of oxygen atoms (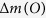) that are near the interface, as all of them have the same substrate (O-terminated Al_2_O_3_(11
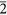
0)). Here, 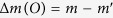, *m* and 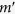 is the Mulliken population for oxygen atoms with and without Nb film, respectively. We noted that 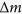 is an average value, as there are more than one equivalent position for oxygen atoms in Al_2_O_3_(11
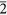
0) plane.

#### ORs of Nb(110)/Al_2_O_3_(11 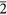 0) interface

The calculation results for the six types of potential models are shown in Table 1. It shows that model b1 has the biggest work of separation (0.77 eV/Å^2^) and the biggest 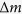 (0.26 e) among the six possible models. This indicates that among all the six potential candidates, the model b1 has the most stable interface structure, and more electrons are transferred from Nb film to Al_2_O_3_ substrate in this model than that occurred in other five models, implying stronger chemical bonds are formed along the interface of the model b1.

**Figure 6:**
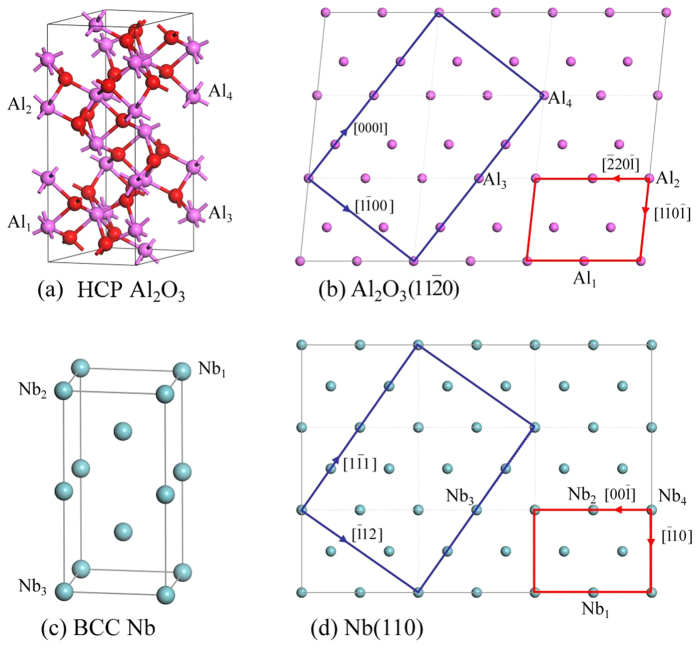
The crystal structures of HCP Al_2_O_3_ and BCC Nb, with the indications of atomic arrangements in Al_2_O_3_(11
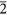
0) and Nb(110). (**a**,**c**) The atomic structure of Al_2_O_3_ and Nb. (**b**) The atomic lattice of Al_2_O_3_(11
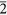
0) plane, and (**d**) The corresponding match plane Nb(110).

The detailed lattice structure of the model b1 is shown in Fig. 6. The model b1 of the interface is constructed by two unit cells: the primitive unit cell of Al_2_O_3_(11
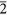
0), and a rectangular unit cell of Nb(110), with the ORs of Nb[00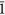]//Al_2_O_3_[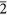
20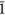] (*l* direction), Nb[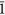
10]//Al_2_O_3_[1
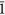
0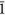] (*m* direction) and Nb[110]//Al_2_O_3_[11
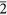
0] (*n* direction), as the red cells shown in Fig. 6b,d. The lattice strain (ε = (a′ − a)/a) for the Nb cell along *l* and *m* directions is (6%, 10%).

Now we compare the lattice mismatch between the present simulation and experiment. The ORs of Nb(110)//Al_2_O_3_(11
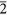
0) observed in the experiment are Nb[1
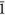
1]// Al_2_O_3_[0001], Nb[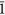
12]// Al_2_O_3_[1
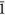
00], and Nb(110)// Al_2_O_3_(11
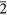
0), which look different from the present simulation model. However, if we expand the simulation model by three times in *l* and *m* directions, we can construct a supercell which is the same as experiments (blue cells in both Al_2_O_3_ and Nb plane), as shown in Fig. 6b,d. The blue cell in Al_2_O_3_(11
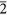
0) is 12.991 (along [0001]) × 8.243 (along [1
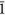
00]) Å^2^, while the blue cell in Nb(110) is 11.421(along [1
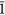
1]) × 8.076 (along [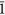
12]) Å^2^. Thus the lattice mismatch (δ = 2|(d_s_ − d_f_)|/(d_s_ + d_f_)) along Nb[1
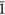
1] and Nb[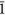
12] directions is: 12.9% and 2.0% respectively. These calculated values are very close to the observed data in experiment (12.5%, 1.8%). As a result, it is concluded that the present simulation model explains the experimental results.

Interestingly, we also noticed from Table 1 that the smallest mismatch at interface does not naturally relate to the interface with the biggest work of separation (the strongest chemical bound), e.g., the result of the model b2. In contrast, model b1 has the largest 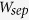 while the interface mismatch is not the smallest (δ′(b2) < δ′(b1)). As both TEM and XRD results confirmed that the interfacial configuration of Nb/Al_2_O_3_(11
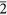
0) favors model b1, this indicates that although misfit is important for the interface structure of metal/oxide system, the work of separation and the formation of chemical bound may be the dominant factors that determine the ORs between the metal/oxide system.

#### Comparison of interfaces in Nb/Al_2_O_3_ and Nb/MgO systems

The present experiments have shown that crystalline quality of Nb on Al_2_O_3_ is better than that on MgO. To understand the underlying reason, we further build the coherent structures of Nb(110)/MgO(111), and repeat the calculating procedures as shown for Nb/Al_2_O_3_(11
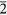
0), and then make a comparison of the two lattice structures. It should be noted that here we calculated the difference of Mulliken population of Nb atoms (Δm(Nb)), as both the Nb(110)/Al_2_O_3_(11
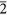
0) and Nb(110)/MgO(111) structure have the same Nb(110) film.

The calculated results are listed in Table 2. First, it is noticed that the interface mismatch (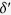) of Nb(110)/Al_2_O_3_(11
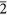
0) system is 0.07 eVÅ^−2^, smaller than that in Nb(110)/MgO(111) system (0.10 eVÅ^−2^). This indicates that there is a better interface overlap of Nb/Al_2_O_3_ than that of Nb/MgO. Meanwhile, the lattice mismatch for Nb(110)/Al_2_O_3_ (11
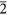
0) at *l* and *m* direction (2.0%, 12.9%) is also smaller than that of Nb(110)/MgO(111) (10.0%, 10.2%). A small lattice mismatch means a large distance between misfit dislocations along the interface of Nb/Al_2_O_3_, which is consistent with the dislocation spacing observed in the experiment. Second, it is noticed that the work of separation of Nb(110)/Al_2_O_3_(11
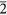
0) system (0.77 eV/Å^2^) is larger than that of Nb(110)/MgO(111) system (0.74 eV/Å^2^). It means the combined strength between Nb film and Al_2_O_3_ substrate is stronger, and defects might be more difficult to form along the interface in this system. In addition, Table 2 also showed that Nb atoms in Nb(110)/Al_2_O_3_(11
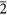
0) system lose more electrons (0.47 e) than that in Nb(110)/MgO(111) system (0.25 e). This indicates that stronger ionic bonds are formed along the interface Nb/Al_2_O_3_ system. In summary, it is concluded that the higher degree of crystal perfection of Nb(110)/Al_2_O_3_(11
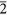
0) likely arises from the smaller interface mismatch, larger work of separation and stronger ionic bonds.

**Figure 7:**
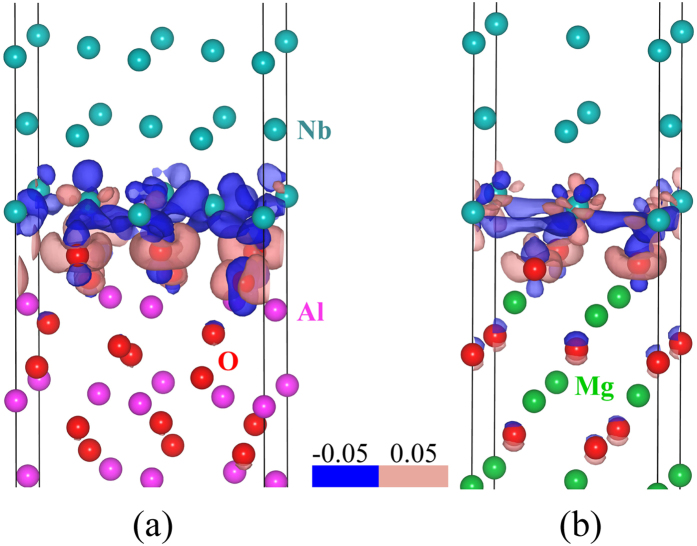
Electron density map of (**a**) Nb(110)/Al_**2**_**O**_**3**_**(11**
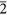
0) and (**b**) Nb(110)/MgO(111) interfaces. The isosurface value for the light red color (electrons accumulation) is 0.05 electrons/Å^3^, and for dark blue color (electrons depletion) is −0.05 electrons/Å^3^. Color code of the atoms: Al (violet), O (red), Nb(blue), Mg(green).

The bonding information can also be visualized by delocalization of atomic charge density. The electron density difference (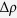) maps of the above two interface structures are shown in Fig. 7, where 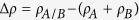, 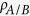 is the self-consistent electron density of whole relaxed metal/substrate interface structure, 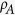 and 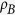 are the electron densities of isolated metal and substrate slabs having the same atomic positions in the interface structure. We observed from Fig. 7 that for both systems, Nb atoms lose electrons, and O atoms gain electrons, as the blue and light red color shown. This means electrons are transferred from Nb film to substrate along the interface, and the transfer mainly takes place between the two adjacent layers next to the interface. However, the electrons redistribution in Nb(110)/Al_2_O_3_(11
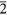
0) is stronger than that in Nb(110)/MgO(111). This indicates that ionic bonds in Nb/Al_2_O_3_ are stronger than that in Nb/MgO system. In fact, the electron density difference maps can also be understood as follow: the number of electron suppliers are almost the same Nb(110) in both systems, however, the atomic ratio of the electron supplier(Nb) and electron acceptor (oxygen) is 2:3 in the Nb/Al_2_O_3_ system, while this value for Nb/MgO system is 1:1. Thus it is natural that the Nb atoms in Nb/Al_2_O_3_ interface will lose more electrons than that in Nb/MgO system.

In conclusion, Nb films were deposited on the Al_2_O_3_(11
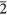
0) substrate and MgO(111) substrate by e-beam evaporation method. The interface structures of these two interfaces were investigated by the experiments and simulations. ORs of Nb/Al_2_O_3_(11
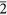
0) interface have been confirmed as Nb(110)//Al_2_O_3_(11
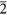
0)and Nb[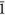
12]//Al_2_O_3_[1
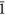
00], and the ORs of Nb/MgO(111) interface are Nb(110)//MgO(111) and Nb[001]//MgO[1
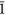
0]. Comparison study shows that the degree of Nb crystalline perfection in Nb(110)/Al_2_O_3_ (11
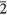
0) system is higher than that in Nb(110)/MgO(111). Frist-principle calculations through a coherent interface model showed that the higher crystal perfection of Nb(110)/Al_2_O_3_(11
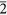
0) arises from the smaller interface mismatch, larger work of separation and stronger ionic bonds. Although misfit is important for forming the interface configuration of metal/oxide interface system, the work of separation and the strength of chemical bound may be the dominant factors that determine the interface configuration in the metal/oxide interface system.

## Methods

### Sample preparation and characterization

Nb thin films were deposited on single crystal Al_2_O_3_(11
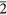
0) substrate and MgO(111) substrate by electron-beam evaporation technique with a deposition rate of 5Å/s at 950 °C under a vacuum of 3.5 × 10^−5^ Pa. The purity of the Nb evaporation target was 99.999%. The thickness of Nb thin film is 170 nm. The single crystal Al_2_O_3_ substrate has the dimension of 10 mm × 10 mm × 0.5 mm. The detailed deposition conditions of Nb film on single crystal MgO(111) can be found elsewhere^27^. XRD analysis was used to characterize the Nb film structure and the ORs between the Nb film and the substrates. The cross sectional TEM samples were prepared at room temperature by using conventional thinning-milling process. The FEI Tecnai F30 transmission electron microscope (TEM), which worked at 300 kV with a field-emission gun, was used to characterize the interface structure of Nb film on different substrates. The selected area electron diffraction (SAD) was used to identify the ORs between the Nb films and the substrates. RBS/Channeling measurement was used to characterize the crystal structure of deposited Nb films. The analyzing beam was 2 MeV ^4^He^+^ ions and the beam current was about 10 nA with an area of 1 mm^2^.

## Additional Information

**How to cite this article**: Du, J. L. *et al*. What determines the interfacial configuration of Nb/Al_2_O_3_ and Nb/MgO interface. *Sci. Rep*. **6**, 33931; doi: 10.1038/srep33931 (2016).

## Supplementary Material

Supplementary Information

## Figures and Tables

**Table 1:** Structure parameters and calculation results of the six Nb/Al_2_O_3_(11
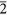
0) coherent interface models (model b1–b6 in Figure S1b), including orientation relationships (ORs), interface misfit (δ′), lattice strain of Nb cell (*ε*), work of separation (*W*_*sep*_, given in eVÅ^−2^), and the difference in atomic Mulliken population of oxygen atoms (Δ*m*(O), given in electrons).

Model	ORs	lattice strain (*ε*_*l*_, *ε*_*m*_, *ε*_*n*_)	interface mismatch δ′	*W*_*sep*_	Δ*m*(O)
b1	Nb(110)/Al_2_O_3_(11 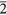 0) Nb[00 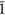 ]//Al_2_O_3_[ 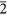 20 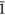 ] Nb[ 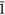 10]//Al_2_O_3_[1 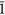 0 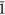 ]	(6%, 10%, −8%)	7.0%	0.77 eVÅ^−2^	0.26
b2	Nb(110)/Al_2_O_3_(11 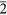 0) Nb[3 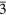 1]//Al_2_O_3_[ 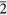 20 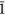 ] Nb[ 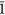 1 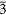 ]//Al_2_O_3_[1 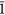 0 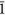 ]	(−3%, −6%, 6%)	4.0%	0.72 eVÅ^−2^	0.25
b3	Nb(110)/Al_2_O_3_(11 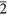 0) Nb[1 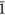 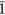 ]//Al_2_O_3_[1 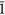 02] Nb[1 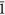 1]//Al_2_O_3_[1 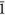 00]	(6%, −4%, −1%)	5.0%	0.63 eVÅ^−2^	0.22
b4	Nb(100)/Al_2_O_3_(11 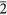 0) Nb[011]//Al_2_O_3_[1 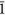 02] Nb[0 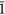 2]//Al_2_O_3_[1 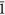 00]	(−3%, 12%, −5%)	7.0%	0.56 eVÅ^−2^	0.19
b5	Nb(111)/Al_2_O_3_(11 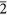 0) Nb[1 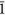 0]//Al_2_O_3_[1 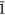 02] Nb[10 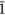 ]//Al_2_O_3_[1 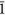 00]	(−3%, −12%, 5%)	8.0%	0.45 eVÅ^−2^	0.16
b6	Nb(111)/Al_2_O_3_(11 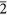 0) Nb[1 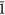 0]//Al_2_O_3_[1 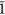 02] Nb[11 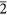 ]//Al_2_O_3_[1 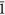 00]	(−3%, 2%, 5%)	8.0%	0.48 eVÅ^−2^	0.18

**Table 2:** Comparison between Nb(110)/Al_2_O_3_(11
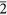
0) and Nb(110)/MgO(111) interfaces, including orientation relationships (ORs), lattice mismatch along *l* and *m* directions ((δ_*l*_, δ_*m*_), both simulation and experiment values), interface misfit (δ′), work of separation (*W*_*sep*_, given in eVÅ^−2^), and the difference in atomic Mulliken population of Nb atoms (Δ*m*(Nb), given in electrons).

Model	ORs (*l, m, n*)	lattice mismatch (δ_*l*_, δ_*m*_)	interface mismatch δ′	*W*_*sep*_	Δ*m*(Nb)
Nb/Al_2_O_3_	Nb[1 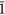 1]//Al_2_O_3_[0001] Nb[ 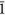 12]//Al_2_O_3_[1 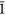 00] Nb[110]//Al_2_O_3_[11 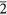 0]	Sim: (12.9%, 2.0%) Exp: (12.5%, 1.8%)	7.0%	0.77 eVÅ^−2^	−0.47
Nb/MgO	Nb[001]//MgO[1 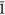 0] Nb[1 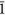 0]//MgO[11 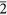 ] Nb[110]//MgO[111]	Sim: (10.0%, 10.2%) Exp: (10.1%, 10.2%)	10.0%	0.74 eVÅ^-2^	−0.25
