# Supplementary material for: What determines the interfacial configuration of Nb/Al2O3 and Nb/MgO interface
Source: Sci Rep. 2016 Oct 4;6:33931. doi: 10.1038/srep33931 (PMC5048433; doi:10.1038/srep33931)
Supplement: Supplementary Information [file srep33931-s1.doc]

**What determines the interfacial configuration of Nb/Al_2_O_3_ and Nb/MgO interface**

J.L. Du ^1^, Y. Fang ^2^, E.G. Fu ^1,*^ , X. Ding ^2,*^, K.Y. Yu ^3,*^, Y.G. Wang ^1^, Y.Q. Wang ^4^, J.K. Baldwin ^4^, P.P. Wang ^1^, Q. Bai ^1^

1. State Key Laboratory of Nuclear Physics and Technology, School of Physics, Peking University, Beijing 100871, P. R. China
2. State Key Laboratory for Mechanical Behavior of Materials, Xi’an Jiaotong University, Xi’an 710049, P. R. China
3. Department of Materials Science and Engineering, China University of Petroleum, Beijing 102249, P. R. China
4. Experimental Physical Sciences Directorate, Los Alamos National Laboratory, Los Alamos, NM 87544, USA

**Support Information**

Simulation model and method

In current first-principle studies, two complementary approaches are extensively used for quantum mechanical modeling of interface: a small cluster of atoms and a periodic slab of crystal layers [1]. The cluster model is widely used to accurately provide the adsorption energy and bounding strength of adatoms or clusters deposited on oxide surfaces. The periodic slab model, which could be divided into coherent and incoherent periodic models, represents an interface as a sandwich of semi-infinite crystal layers and can take account of the metallic binding character.

Hashibon et al. [2] pointed out that the surface relaxation does not alter the hierarchy of the work of separation, and thus coherent periodic model is sufficient to compare the stability of various interfaces. Here we use the coherent periodic slab model to understand the interface structures of Nb/Al_2_O_3_(11$\bar{2}$0) and Nb/MgO(111) interfaces and investigate the structure similarities and differences between the above two systems.

The simulation results of lattice constants at 0K for Al_2_O_3_ are *a = b* = 4.759 Å and *c* = 12.991 Å, and for BCC Nb are *a* = 3.297 Å. For Nb/Al_2_O_3_ system, early experiments suggested the monolayer of Nb strongly prefers those surface sites on Al_2_O_3_ substrate that are occupied by Al in the bulk [1], and the final layer on the Al_2_O_3_ side of Nb/Al_2_O_3_ interface is composed of oxygen atoms [3]. Thus, the O-terminated Al_2_O_3_(11$\overline{2}$0) is used as the substrate to build the Nb/Al_2_O_3_(11$\overline{2}$0) coherent interface model, and the sites of Nb are set on the Al_2_O_3_ substrate that are occupied by Al atom in the bulk. Meanwhile, for the given O-terminated Al_2_O_3_(11$\overline{2}$0) substrate, theoretically there could be an infinite number of match types for the Nb plane matching the Al_2_O_3_(11$\overline{2}$0) plane. However, metal/ceramic interface shows that the special orientations are such that low-indexed planes and directions of one crystal lie parallel to low-indexed planes and directions of another crystal [4, 5-7]. Therefore, three low-indexed planes of Nb film are considered for simplicity, and they are Nb(100), Nb(110) and Nb(111).

For a given Nb/Al_2_O_3_ coherent model, e.g., Nb(110)//Al_2_O_3_(11$\bar{2}$0), there are still infinite ways to match these two parallel planes. The “interface builder” tool box in Atomistix toolKit (ATK) software [1, 8, 9] was used to search the possible models, and the process is shown in Figure S1(a). Based on the consideration of both the maximum atomic number and proper computation time, we set an upper limit of lattice strain ($\varepsilon$) of Nb layer and interface mismatch ($\delta'$) to reduce the number of possible candidates. Lattice strain is the strain of Nb along a given direction, e.g., as $x$, where $\Delta m$ and $W_{\text{sep}}$ are the lattice constants after and before deformation along *l* direction. The interface mismatch which is thought to be the two-dimensional strain during the coherent structure formation of two unit cells, is defined as $\overline{2}$,where $\overline{2}$ represents the overlap area of unit cell $\overline{2}$ with the match cell area of $\overline{2}$ and the unit cell $\overline{2}$ with the match area of $\overline{2}$[10]. It is noted that $\delta'$ indicates the mismatch between two unit cells, which differs from the lattice mismatch in one direction (δ). For the Nb/ Al_2_O_3_(11$\bar{2}$0) system, the cutoff values of interface mismatch $\delta'$, lattice strain $\varepsilon$ (among l, m, n direction) of Nb are set as $\delta'$ = 12%, and $\varepsilon_{l}$ = 12%. With the above criteria, we can reduce the possible coherent models to 3 types of Nb(110)/Al_2_O_3_(11$\varepsilon_{l}$0). They are indicated in Fig. S1(a) by the three dark gray balls, and these models are shown in Fig. S1(b1), (b2), (b3). By repeating this process, we can also obtain one type of potential coherent model for Nb(100)/Al_2_O_3_(11$\overline{2}$0) (Fig. S1(b4)), and two types of models for Nb(111)/Al_2_O_3_(11$\varepsilon_{m}$0) (Fig.S1(b5), (b6)).

For each coherent model, the layer number of Nb film is set to be not smaller than five, and the layer number of Al_2_O_3_ film is set to be not smaller than six. The thickness of vacuum layer is set to 15 Å. The structures are first optimized by minimizing the total energy of the system, and then the electronic structure calculations are carried out. Density functional theory (DFT) calculating engine in ATK was employed to do all calculations [1, 8, 9]. Spin-polarized local density approximation (LSDA) with the Perdew-Zunger parameterization was used as the exchange and correlation function [11, 12]. Single zeta plus polarization basis set was used for the electron wave function. A cutoff energy of 75 Hartree and a Monkhorst-Pack k-mesh of 7×7×1 were used for electronic structure and total energy calculations.

Reference

[1] Ernst, F. Metal-Oxide Interface. *Materials Science and Engineering R* **14,** 97-156 (1995).

[2] Brandbyge, M.; Mozos, J.L.; Ordejón, P.; Taylor, J.; Stokbro, K. Self-interaction Correction to Density Functional Approximations For Many-electron Systems. *Physical Review B* **65,** 16 (2002).

[3] Demkowicz, M.J., Misra, A.; Caro, A. The Role of Interface Structure in Controlling High Helium Concentrations. *Current Opinion in Solid and Materials Science* **16,** 101-108 (2012).

[4] Fu, E.G. *et al.* Interface Structure of Nb Films on Single Crystal MgO(100) and MgO(111) Substrates. *Acta Materialia* **64,** 100-112 (2014).

[5] Wang, C.M.; Kaspar, T.C.; Shutthanandan, V.; Joly, A.G.; Kurtz, R.J. Structure of Cr Film Epitaxially Grown on MgO(001). *Acta Materialia* **59,** 4274-4282 (2011).

[6] Kuwabara, M.; Spence, J.C.H.; Ruhle, M. On the Atomic Structure of the Nb/Al_2_O_3_ Interface and the Growth of Al_2_O_3_ Particles. *Journal of Materials Research* **4,** 972-977 (1989).

[7] Ikuhara, Y.; Sugawara, Y.; Tanaka. I. Atomic and Electronic Structure of V/MgO Interface. *Interface Science* **5,** 5-16 (1997).

[8] QuantumWise, Atomistix ToolKit(ATK) http://www.quantumwise.com.

[9] Soler, J.M. *et al.* The SIESTA Method for ab Initio Order–*N* Materials Simulation. *Journal of Physics-Condensed Matter* **14,** 2745-2779 (2002).

[10] Grier, E.J.; Jenkins, M.L.; Petford-Long, A.K.; Ward, R.C.C.; Wells. M.R. Misfit Dislocations of Epitaxial (110) Niobium Parallel to (11$\overline{2}$0) Sapphire Interfaces Grown by Molecular Beam Epitaxy. *Thin Solid Films* **358,** 94-98 (2000).

[11] Stivastava, G.P.; Weaire，D. The Theory of the Cohesive Energies of Solids. *Advances in Physics* **36,** 463-517 (1987).

[12] Perdew, J.P.；Zunger, A. Self-interaction Correction to Density Functional Approximations for Many-electron Systems. *Physical Review B* **23,** 5048-5079 (1981).


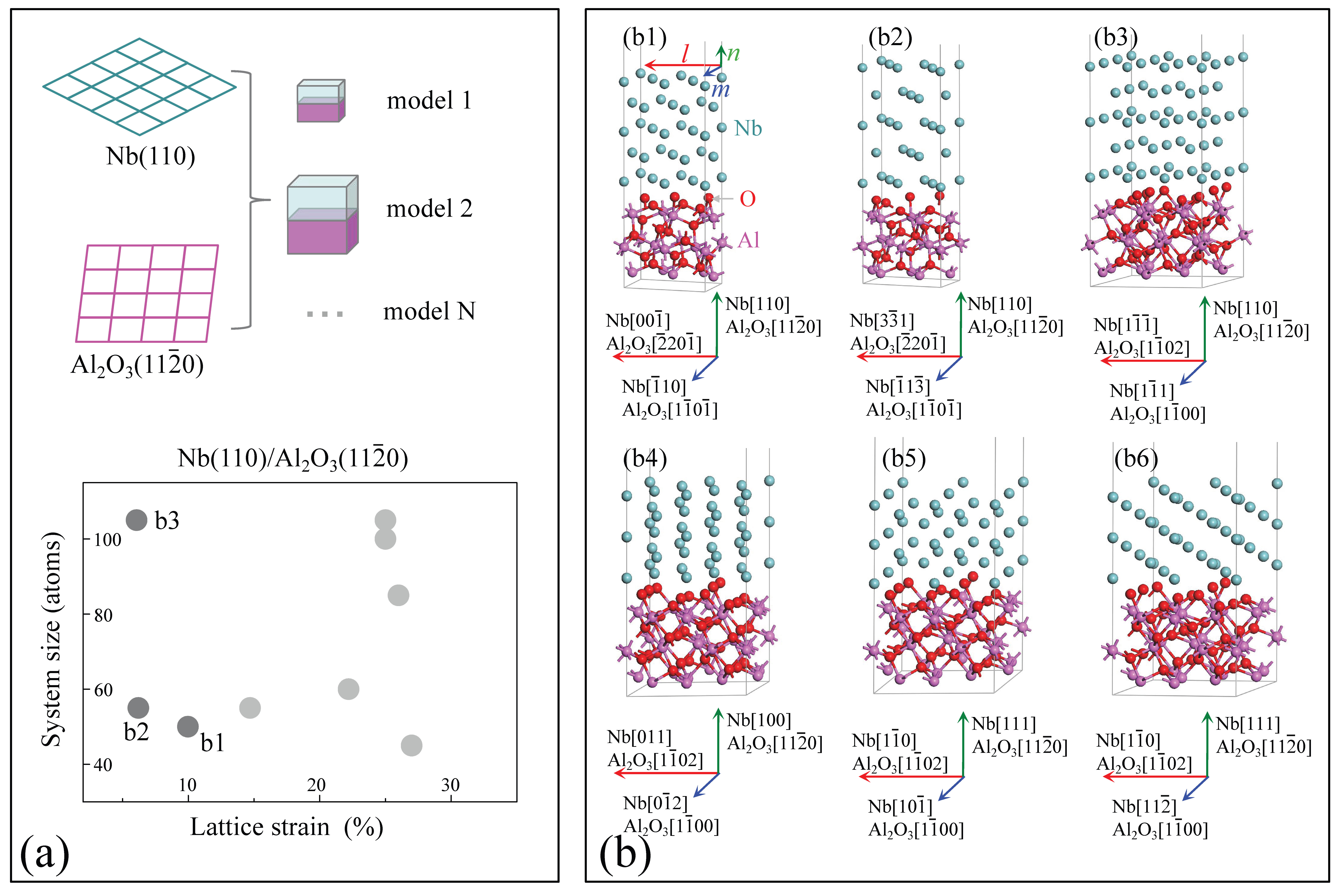


Figure S1. Coherent interface structures of Nb/Al_2_O_3_(11$\overline{2}$0). (a) Buildup of the coherent Nb(110)/Al_2_O_3_(11$\overline{2}$0) interface models by ATK software. (b) Periodic slab model of six types of coherent Nb/Al_2_O_3_(11$\overline{2}$0) interface structures, with the indication of orientation relationships along *l*, *m* and *n* directions. The six types of models are: three types of Nb(110)/Al_2_O_3_(11$\overline{2}$0) models (b1-b3), one type of Nb(100)/Al_2_O_3_(11$\overline{2}$0) model (b4), and two types of Nb(111)/Al_2_O_3_(11$\overline{2}$0) models (b5, b6). Al, O, Nb atoms are indicated by different colors: Al (violet), O (red), Nb(blue).
